# Differential Targeting of Unpaired Bases within Duplex DNA by the Natural Compound Clerocidin: A Valuable Tool to Dissect DNA Secondary Structure

**DOI:** 10.1371/journal.pone.0052994

**Published:** 2012-12-28

**Authors:** Matteo Nadai, Giorgio Palù, Manlio Palumbo, Sara N. Richter

**Affiliations:** 1 Department of Molecular Medicine, University of Padua, Padua, Italy; 2 Department of Pharmaceutical and Pharmacological Sciences, University of Padua, Padua, Italy; University of Quebect at Trois-Rivieres, Canada

## Abstract

Non-canonical DNA structures have been postulated to mediate protein-nucleic acid interactions and to function as intermediates in the generation of frame-shift mutations when errors in DNA replication occur, which result in a variety of diseases and cancers. Compounds capable of binding to non-canonical DNA conformations may thus have significant diagnostic and therapeutic potential. Clerocidin is a natural diterpenoid which has been shown to selectively react with single-stranded bases without targeting the double helix. Here we performed a comprehensive analysis on several non-canonical DNA secondary structures, namely mismatches, nicks, bulges, hairpins, with sequence variations in both the single-stranded region and the double-stranded flanking segment. By analysis of clerocidin reactivity, we were able to identify the exposed reactive residues which provided information on both the secondary structure and the accessibility of the non-paired sites. Mismatches longer than 1 base were necessary to be reached by clerocidin reactive groups, while 1-base nicks were promptly targeted by clerocidin; in hairpins, clerocidin reactivity increased with the length of the hairpin loop, while, interestingly, reactivity towards bulges reached a maximum in 3-base-long bulges and declined in longer bulges. Electrophoretic mobility shift analysis demonstrated that bulges longer than 3 bases (i.e. 5- and 7-bases) folded or stacked on the duplex region therefore being less accessible by the compound. Clerocidin thus represents a new valuable diagnostic tool to dissect DNA secondary structures.

## Introduction

Nucleic acids are highly polymorphic: depending on the sequences and environmental conditions they may exist in a variety of secondary structures such as duplexes, triplexes, tetraplexes, bulges, hairpins, loops [1], [2]. Such non-canonical structures in nucleic acids are of general biological significance: they have been postulated to mediate protein-nucleic acid interactions, either by contacting protein residues directly or by producing a distinct tertiary structure to which the protein binds [3], and to function as intermediates in the generation of frame-shift mutations when errors in DNA replication occur [4], [5]. In particular, extra-helical bases are thought to be implicated in nucleic acid non-canonical functions [6]. An essentially unlimited combination of secondary structural elements has been extensively described in RNA, where the single-stranded (ss) nucleic acid folds back on itself; however, DNA can also produce complex secondary structures during replication and recombination [7]. A shift of the reading frame during template-dependent DNA synthesis can lead to the addition or deletion of one or more nucleotide residues (nts) in the newly synthesized DNA, ensuing in bulged or mismatched structures. Bulged bases derived from replicative errors are considered the first step of frame-shift mutagenesis [6], resulting in a variety of diseases and cancers (e.g., myotonic dystrophy, Huntington's disease, Friederich's ataxia, and fragile X syndrome).

In general, compounds capable of binding to non-canonical conformations of the DNA could have significant therapeutic potential. Several derivatives with unrelated structures have been reported to individually target sequence-specific bulges [8], [9], [10], mismatches [11], [12] and loops [13]. However, it is not clear whether a particular disease is linked to only one sequence-specific DNA conformation; therefore, compounds able to universally target DNA unconventional structures within a duplex section of DNA could be appealing from both a therapeutic and diagnostic point of view.

**Figure 1:**
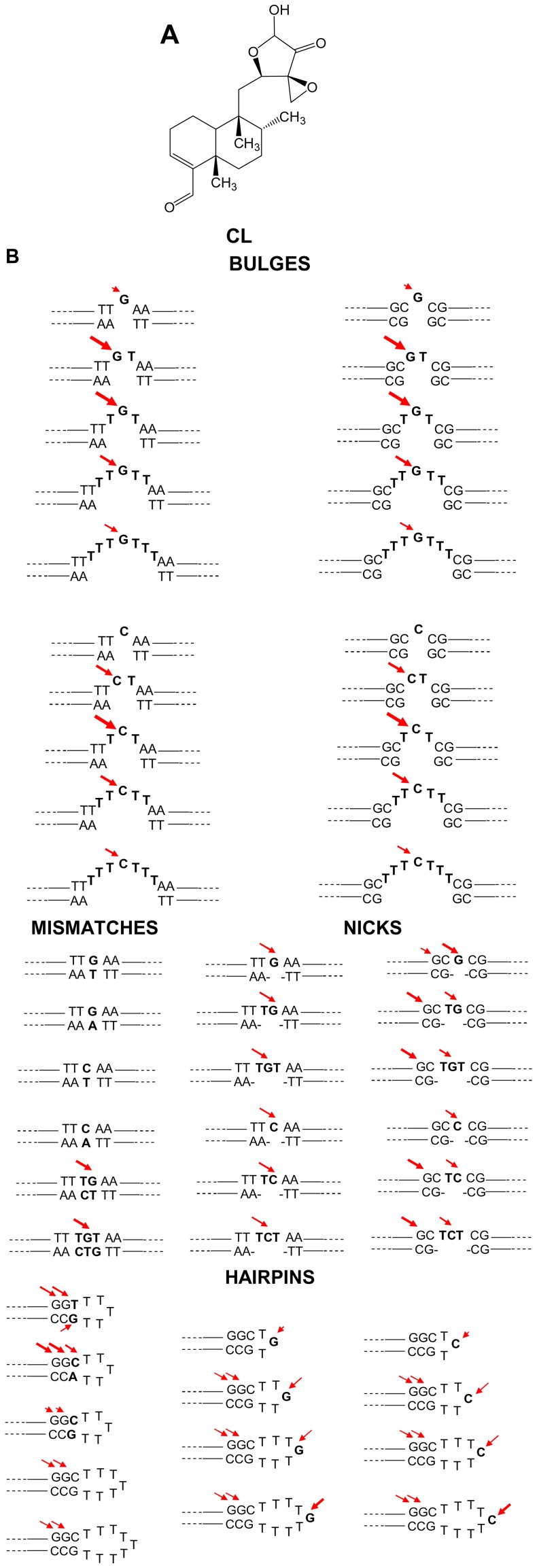
Reagents used in this study. A) Chemical structure of CL. B) Schematic representation of the single-stranded (ss) regions of the oligonucleotides used, subdivided according to the secondary structure category. Double-stranded regions flanking the ss moiety are shown, because CL reactivity was assayed and compared towards oligonucleotides with both G/C and A/T-rich flanking regions. Arrows indicate the position of CL alkylation and cleavage. The size of the arrows corresponds to the degree of reactivity.

Clerocidin (CL) (Fig. 1A) is a natural product isolated from *Oidiodendron truncatum*, initially described as a gyrase inhibitor [14], [15], [16]. CL was subsequently shown to target DNA ss regions while being unreactive towards the double-helix: CL directly reacts with three-base DNA bulges, with different mechanisms depending on the exposed nucleotide. In particular, CL electrophilic groups (i.e. a strained epoxy ring and an α-ketoaldehyde function in equilibrium with its hemi-acetalic form) target i) the nucleophilic N7 of guanine (G) inducing spontaneous depurination and DNA strand cleavage [17], [18], ii) the NH_2_ and N3 of cytosine (C) with formation of a stable condensed ring system, which is degraded to induce DNA cleavage only after hot alkali treatment [19], and iii) the NH_2_ and N1 of adenine (A) to generate an adduct that degrades upon alkali but does not result in DNA strand scission [20]. Due to lack of strong nucleophilic sites, thymine (T) does not react with CL. The bulky diterpenoid portion of CL modulates the accessibility of the epoxide and α-ketoaldehyde reactive groups towards the DNA.

Here we sought to investigate the ability of CL to target bases embedded in different DNA conformational environments, such as mismatched bases and nicked DNA, loops and hairpins. Our results showed that CL was able to react with most ss structures within a duplex DNA; however, the number of ss bases was important to determine the accessibility of the compound to the reactive site. Therefore CL, besides being able to target a wide range of ss structures in a double helix setting, can also be used as a tool to evaluate site accessibility and folding of ss areas of the DNA within a double helix environment.

## Materials and Methods

### Clerocidin and Oligonucleotides

CL was a gift of Leo Pharmaceutical Products (Ballerup, Denmark). Molar extinction coefficients were experimentally determined to be 11818 M^−1^ cm^−1^ for CL. Working drug solutions were obtained by diluting fresh stocks in the appropriate buffer. T4 polynucleotide kinase was purchased from Fermentas (Burlington, Canada). [γ-^32^P]ATP was from Perkin Elmer (MA, USA), while all oligonucleotides were from Sigma Aldrich (St Louis, MO, USA). Oligonucleotides used in this study are reported in Table 1.

**Table 1:** Oligonucleotides used in this study.

Oligonucleotide			
Type	Name	Sequence	Abbreviated description
**Mismatch G**	1	CTATTGCTTTATTT**G**TAACCATTATAAGCT	MM G/T
	1 rev	AGCTTATAATGGTTA**T**AAATAAAGCAATAG	
	1	CTATTGCTTTATTT**G**TAACCATTATAAGCT	MM G/A
	1a rev	AGCTTATAATGGTTA**A**AAATAAAGCAATAG	
	1	CTATTGCTTTATT**TG**TAACCATTATAAGCT	MM TG/TC
	2 rev	AGCTTATAATGGTTA**TC**AATAAAGCAATAG	
	1	CTATTGCTTTATT**TGT**AACCATTATAAGCT	MM TGT/GTC
	3 rev	AGCTTATAATGGTT**GTC**AATAAAGCAATAG	
**Mismatch C**	4	CTATTGCTTTATTT**C**TAACCATTATAGCT	MM C/T
	1 rev	AGCTTATAATGGTTA**T**AAATAAAGCAATAG	
	5	CTATTGCTTTATTT**C**TAACCATTATAAGCT	MM C/A
	1a rev	AGCTTATAATGGTTA**A**AAATAAAGCAATAG	
**Bulge G** **A/T rich**	1	CTATTGCTTTATTT**G**TAACCATTATAAGCT	B1 G A/T rich
	1b rev	AGCTTATAATGGTTAAAATAAAGCAATAG	
	1	CTATTGCTTTATTT**GT**AACCATTATAAGCT	B2 G A/T rich
	1c rev	AGCTTATAATGGTTAAATAAAGCAATAG	
	1	CTATTGCTTTATT**TGT**AACCATTATAAGCT	B3 G A/T rich
	1d rev	AGCTTATAATGGTTAATAAAGCAATAG	
	6	CTATTGCTTTATT**TTGTT**AACCATTATAAGCT	B5 G A/T rich
	1d rev	AGCTTATAATGGTTAATAAAGCAATAG	
	7	CTATTGCTTTATT**TTTGTTT**AACCATTATAAGCT	B7 G A/T rich
	1d rev	AGCTTATAATGGTTAATAAAGCAATAG	
**Bulge G** **G/C rich**	8	CTATTGCTTTACGC**G**CGCCCATTATAAGCT	B1 G G/C rich
	8 rev	AGCTTATAATGGGCGGCGTAAAGCAATAG	
	9	CTATTGCTTTCGC**TG**CGCCCATTATAAGCT	B2 G G/C rich
	9 rev	AGCTTATAATGGGCGGCGTAAAGCAATAG	
	10	CTATTGCTTTCGC**TGT**CGCCATTATAAGCT	B3 G G/C rich
	10 rev	AGCTTATAATGGCGGCGAAAGCAATAG	
	11	CTATTGCTTTCGC**TTGTT**CGCCATTATAAGCT	B5 G G/C rich
	10 rev	AGCTTATAATGGCGGCGAAAGCAATAG	
	12	CTATTGCTTTCGC**TTTGTTT**CGCCATTATAAGCT	B7 G G/C rich
	10 rev	AGCTTATAATGGCGGCGAAAGCAATAG	
**Bulge C** **A/T rich**	5	CTATTGCTTTATTT**C**TAACCATTATAAGCT	B1 C A/T rich
	1b rev	AGCTTATAATGGTTAAAATAAAGCAATAG	
	1	CTATTGCTTTATTT**CT**AACCATTATAAGCT	B2 C A/T rich
	1c rev	AGCTTATAATGGTTAAATAAAGCAATAG	
	5	CTATTGCTTTATT**TCT**AACCATTATAAGCT	B3 C A/T rich
	1d rev	AGCTTATAATGGTTAATAAAGCAATAG	
	13	CTATTGCTTTATT**TTCTT**AACCATTATAAGCT	B5 C A/T rich
	1d rev	AGCTTATAATGGTTAATAAAGCAATAG	
	14	CTATTGCTTTATT**TTTCTTT**AACCATTATAAGCT	B7 C A/T rich
	1d rev	AGCTTATAATGGTTAATAAAGCAATAG	
**Bulge C** **G/C rich**	17	CTATTGCTTTACGC**C**CGCCCATTATAAGCT	B1 C G/C rich
	8 rev	AGCTTATAATGGGCGGCGTAAAGCAATAG	
	18	CTATTGCTTTCGC**TC**CGCCCATTATAAGCT	B2 C G/C rich
	9 rev	AGCTTATAATGGGCGGCGAAAGCAATAG	
	19	CTATTGCTTTCGC**TCT**CGCCATTATAAGCT	B3 C G/C rich
	10 rev	AGCTTATAATGGCGGCGAAAGCAATAG	
	20	CTATTGCTTTCGC**TTCTT**CGCCATTATAAGCT	B5 C G/C rich
	10 rev	AGCTTATAATGGCGGCGAAAGCAATAG	
	21	CTATTGCTTTCGCT**TTCTT**TCGCCATTATAAGCT	B7 C G/C rich
	10 rev	AGCTTATAATGGCGGCGAAAGCAATAG	
**Nick G** **A/T rich**	1	CTATTGCTTTATTT**G**TAACCATTATAAGCT	N1 G A/T rich
	11 rev	AGCTTATAATGGTTA	
	12 rev	AAATAAAGCAATAG	
	1	CTATTGCTTTATT**TG**TAACCATTATAAGCT	N2 G A/T rich
	11 rev	ATTGGTAATATTCGA	
	13 rev	AATAAAGCAATAG	
	1	CTATTGCTTTATT**TGT**AACCATTATAAGCT	N3 G A/T rich
	14 rev	AGCTTATAATGGTT	
	13 rev	GATAACGAAATAA	
**Nick G** **G/C rich**	8	CTATTGCTTTACGC**G**CGCCCATTATAAGCT	N1 G G/C rich
	15 rev	AGCTTATAATGGGCG	
	16 rev	GCGTAAAGCAATAG	
	22	CTATTGCTTTCCG**TG**CGCCCATTATAAGCT	N2 G G/C rich
	17 rev	GCGGGTAATATTCGA	
	18 rev	CGGAAAGCAATAG	
	23	CTATTGCTTTCCG**TGT**GCCCATTATAAGCT	N3 G G/C rich
	19 rev	AGCTTATAATGGGC	
	18 rev	GATAACGAAAGGC	
**Nick C** **A/T rich**	24	CTATTGCTTTATTT**C**TAACCATTATAAGCT	N1 C A/T rich
	11 rev	ATTGGTAATATTCGA	
	12 rev	GATAACGAAATAAA	
	24	CTATTGCTTTATT**TC**TAACCATTATAAGCT	N2 C A/T rich
	11 rev	ATTGGTAATATTCGA	
	13 rev	GATAACGAAATAA	
	24	CTATTGCTTTATT**TCT**AACCATTATAAGCT	N3 C A/T rich
	14 rev	TTGGTAATATTCGA	
	13 rev	GATAACGAAATAA	
**Nick C** **G/C rich**	17	CTATTGCTTTACGC**C**CGCCCATTATAAGCT	N1 C G/C rich
	15 rev	-GCGGGTAATATTCGA	
	16 rev	GATAACGAAATGCG-	
	25	CTATTGCTTTCCG**TC**CGCCCATTATAAGCT	N2 C G/C rich
	17 rev	GCGGGTAATATTCGA	
	18 rev	GATAACGAAAGGC	
	26	CTATTGCTTTCCG**TCT**GCCCATTATAAGCT	N3 C G/C rich
	19 rev	CGGGTAATATTCGA	
	18 rev	GATAACGAAAGGC	
**Hairpin T**	27	GCTGCAGCTGG**C**TTTTT**G**CCAGCTGCAGC	H5 T C/G
	28	GCTGCAGCTGG**T**TTTTT**G**CCAGCTGCAGC	H5 T T/G
	29	GCTGCAGCTGG**C**TTTTT**A**CCAGCTGCAGC	H5 T C/A
	30	GCTGCAGCTGG**C**TTTTTTT**G**CCAGCTGCAGC	H7 T C/G
	31	GCTGCAGCTGG**C**TTTTTTTTT**G**CCAGCTGCAGC	H9 T C/G
**Hairpin G**	37	GCTGCAGCTGGCTGTGCCAGCTGCAGC	H3 G
	38	GCTGCAGCTGGCTTGTTGCCAGCTGCAGC	H5 G
	39	GCTGCAGCTGGCTTTGTTTGCCAGCTGCAGC	H7 G
	40	GCTGCAGCTGGCTTTTGTTTTGCCAGCTGCAGC	H11 G
**Hairpin C**	41	GCTGCAGCTGGCTCTGCCAGCTGCAGC	H3 C
	42	GCTGCAGCTGGCTTCTTGCCAGCTGCAGC	H5 C
	43	GCTGCAGCTGGCTTTCTTTGCCAGCTGCAGC	H7 C
	44	GCTGCAGCTGGCTTTTCTTTTGCCAGCTGCAGC	H11 C
**Bulge EMSA**	49	GAAGCTCGC**G**CGCCAGAAG	B1 G short
	49 rev	CTTCTGGCGGCGAGCTTC	
	48	GAGCTCGC**TG**CGCCAGAAG	B2 G short
	48 rev	CTTCTGGCGGCGAGCTC	
	47	GAGCTCGC**TGT**CGCCAGAG	B3 G short
	47 rev	CTCTGGCGGCGAGCTC	
	46	AGCTCGC**TTGTT**CGCCAGA	B5 G short
	46 rev	TCTGGCGGCGAGCT	
	45	GCTCGC**TTTGTTT**CGCCAG	B7 G short
	45 rev	CTGGCGGCGAGC	
	50	GAAGCTCGCGCGCCAGAAG	B ds short
	50 rev	CTTCTGGCGCGCGAGCTTC	
**Hairpin EMSA**	54	CGGCATGCGTGGC**TGT**GCCACGCATGCCG	H3 G short
	53	CGGCAGCGTGGC**TTGTT**GCCACGCTGCCG	H5 G short
	52	CGCAGCGTGGC**TTTGTTT**GCCACGCTGCG	H7 G short
	51	CGCAGCTGGC**TTTTGTTTT**GCCAGCTGCG	H9 G short
	55	CGGCATGCGTGGCT	H ds short
	55 rev	AGCCACGCATGCCG	

### Alkylation by Clerocidin of Single Stranded Bases in a Duplex Environment

All oligonucleotides were gel-purified before use and prepared in desalted/lyophilised form. Forward oligonucleotides were 5′-end-labelled with [γ- ^32^P]ATP by T4 polynucleotide kinase and were subsequently purified by MicroSpin G-25 columns (Amersham Biosciences, Europe). Ss-duplex substrates were obtained by annealing the labelled forward oligonucleotide with equimolar amounts of the reverse, partially complement cold oligonucleotide (Table 1). Drug reactions with the labelled ss-duplex (5 pmol/sample) were performed at 37°C in 50 mM phosphate buffer, pH 7.4. These conditions were selected to maintain the stability of the target oligonucleotide structures and to minimize the possible competition of buffer molecules for drug alkylation [21]. At the indicated time intervals, samples were precipitated with ethanol to eliminate non-reacted drug, then resuspended and either kept on ice, or treated at 90°C for 30 min with 1 M piperidine to complete strand scission according to the Maxam and Gilbert protocol [22]. Samples were then lyophilised, resuspended in formamide gel loading buffer, and heated at 95°C for 3 min. Reaction products were analyzed on 20% denaturing polyacrylamide gels. Gels were visualized by phosphorimaging analysis (Typhoon FLA 9000, GE Healthcare, Europe).

### Electrophoretic Mobility Shift Assay

Labelled ss-duplex substrates, obtained by annealing the labelled forward oligonucleotide with equimolar amounts of the reverse, partially complement cold oligonucleotide (Table 1), were loaded on 12% non denaturing polyacrylamide gels, run for ∼ 7 hours at 75 volts. Gels were visualized by phosphorimaging.

## Results

### Oligo Design and Result Reading

CL (Fig. 1A) was tested against a series of oligonucleotides containing ss regions embedded in a ds environment. Only one single reactive base (either G or C) was positioned in each case in the ss portion. The unreactive T was used when additional ss bases were necessary to obtain the final conformation. The ss motif was flanked alternatively by A/T rich or G/C rich sequences to analyse their effect on the accessibility of the reactive ss site (Table 1).

Reactions were analysed both before and after hot piperidine treatment. Before alkali, alkylation at G bases was normally detected by two bands: one migrating slower than the full-length oligo, indicating alkylation with no DNA cleavage, and one migrating slower than the corresponding G base obtained with the Maxam & Gilbert sequencing reaction, indicating DNA cleavage at G with maintenance of the alkylation adduct. At C, just the band running slower than the full-length oligo was obtained. After piperidine, alkylation at both G and C was manifested as a cleavage band which migrated as the corresponding base obtained in the marker lane, indicating cleavage at G and C and loss of the alkylating CL molecule.

For quantification purposes, the cleavage band obtained after piperidine treatment, which totals up the effect of alkylation and cleavage by CL, was analysed.

### Mismatches

Mismatches form when one or more bases in the forward and reverse strand do not complement. They derive from mis-incorporation of bases that may occur during DNA replication or recombination, or during repairing of DNA damage.

**Figure 2:**
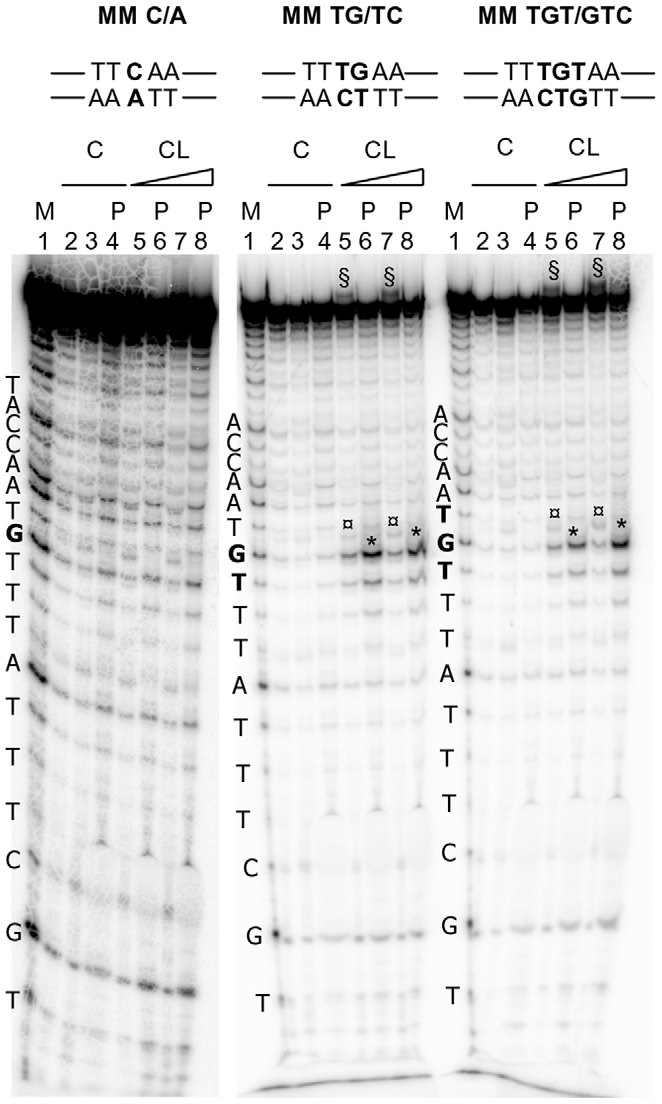
CL footprinting of mismatched oligonucleotides. Oligonucleotides 5, and 1 were heat denaturated and folded in the presence of the appropriate complementary sequences (1a rev, 2 rev, 3 rev, respectively, Table 1) to obtain MM C/A, MM TG/TC and MM TGT/GTC oligonucleotides. The folded oligonucleotides were incubated with increasing concentrations (50–100 µM) of CL for 24 h at 37°C. After reaction, samples were precipitated and either kept on ice or treated with hot piperidine and lyophilized (samples indicated by the symbol P) and loaded on a 20% denaturing polyacrylamide gel. The symbol § indicates CL/full-length DNA adducts which migrate slower than the full-length DNA. The symbol ¤ indicates bands that correspond to the oligonucleotide alkylated and cleaved by CL. CL is still bound to the cleaved oligonucleotide, thus the cleavage band runs slower than the corresponding band in the Maxam and Gilbert marker lane (M lanes). The symbol * indicates bands that correspond to the oligonucleotide alkylated and cleaved by CL, with loss of CL. Position of alkylation is evinced by comparison of cleavage bands after piperidine treatment and the Maxam and Gilbert marker lane. Oligonucleotide sequences are indicated on the left of the corresponding marker lane (M lanes). Base numbering has been assigned in the 5 prime→3 prime direction.

CL was made react with 4 oligonucleotides containing one G or one C base mismatched with T or A, and two oligonucleotides containing TG or TGT bases mismatched with CT or CTG, respectively (Table 1 and Figure 1B). Reaction with the mismatched TG and TGT induced cleavage at the ss G base (before piperidine: symbols ¤ in lanes 5 and 7; after piperidine: asterisks in lanes 6 and 8, Figure 2). These effects increased at higher CL concentration (compare lanes 5 and 7, 6 and 8, Figure 2). Contrary, when the oligonucleotides contained a single mismatched base, no reaction could be observed both before and after piperidine treatment (Figure 2 and Figure 1B for summary).

### Nicks

A nick is a discontinuity in a double stranded DNA molecule where there is no phosphodiester bond between adjacent nucleotides of one strand, typically achieved through damage or enzyme action. Nicks usually release torsion in the strand.

**Figure 3:**
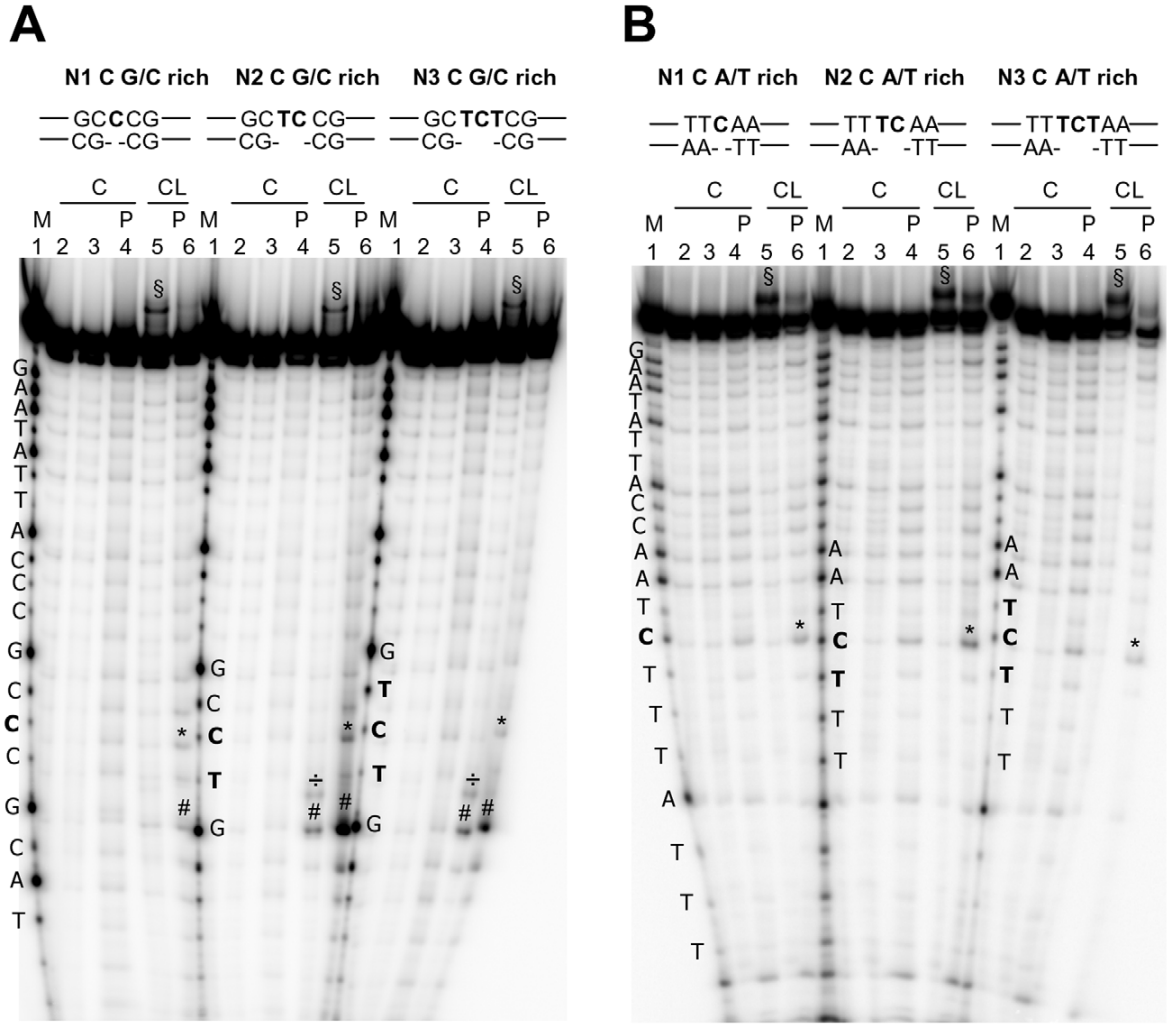
CL footprinting of nicked oligonucleotides. A) Oligonucleotides 17, 25 and 26 were heat denaturated and folded in the presence of the appropriate complementary sequences (15 rev-16 rev, 17 rev-18 rev, 19 rev-18 rev, respectively, Table 1). B) Oligonucleotide 24 was heat denaturated and folded in the presence of the appropriate complementary sequences (12 rev-12 rev, 11 rev-13 rev, 14 rev-13 rev, Table 1) to obtain the nicked C A/T rich oligonucleotides shown above the gel. The folded oligonucleotides were incubated with CL (100 µM) for 24 h at 37°C. After reaction, samples were precipitated and either kept on ice or treated with hot piperidine and lyophilized (samples indicated by the symbol P) and loaded on a 20% denaturing polyacrylamide gel. The symbol § indicates CL/full-length DNA adducts which migrate slower than the full-length DNA. The symbol ¤ indicates bands that correspond to the oligonucleotide alkylated and cleaved by CL. The symbol * indicates bands that correspond to the oligonucleotide alkylated and cleaved by CL, with loss of CL. The symbols ÷ and # indicate bases in the ds region flanking the nicked moiety that are alkylated and cleaved by CL (÷), with loss of CL (#). Position of alkylation is evinced by comparison of cleavage bands after piperidine treatment and the Maxam and Gilbert marker lane. Oligonucleotide sequences are indicated aside of the corresponding marker lane (M lanes). Base numbering has been assigned in the 5 prime→3 prime direction.

Oligonucleotides containing 1, 2 or 3 nicked, non-constrained bases were formed by annealing the forward strand with two partially complementary reverse strands. Each nick contained either one G or C flanked by ss T bases, flanked by A/T- or G/C-rich ds regions (Table 1 and Fig. 1B). Cleavage was modest and comparable between 1-, 2- or 3-base nicks (asterisks, lanes 6, Fig. 3A and B and data not shown). No difference in cleavage intensity was observed between G and C nicked bases and between A/T- and G/C-rich flanking sequences. However, ds Gs close to the nicked portion were cleaved at a higher extent (÷ symbol for slower running bands before piperidine treatment and # symbol for cleavage bands after piperidine, lanes 5 and 6, Fig. 3A and Fig. 1B for summary).

### Bulges

Bulges are formed when bases in one strand have no pairing partner in the opposite strand. They may be created in DNA during recombination between imperfectly homologous sequences and they may exert a role in protein recognition.

Bulges were formed in oligonucleotides containing 1, 2, 3, 5 or 7 non-complemented bases. Each bulge contained either one G or C flanked by ss T bases, adjacent to A/T- or G/C-rich ds regions (Table 1 and Fig. 1B).

**Figure 4:**
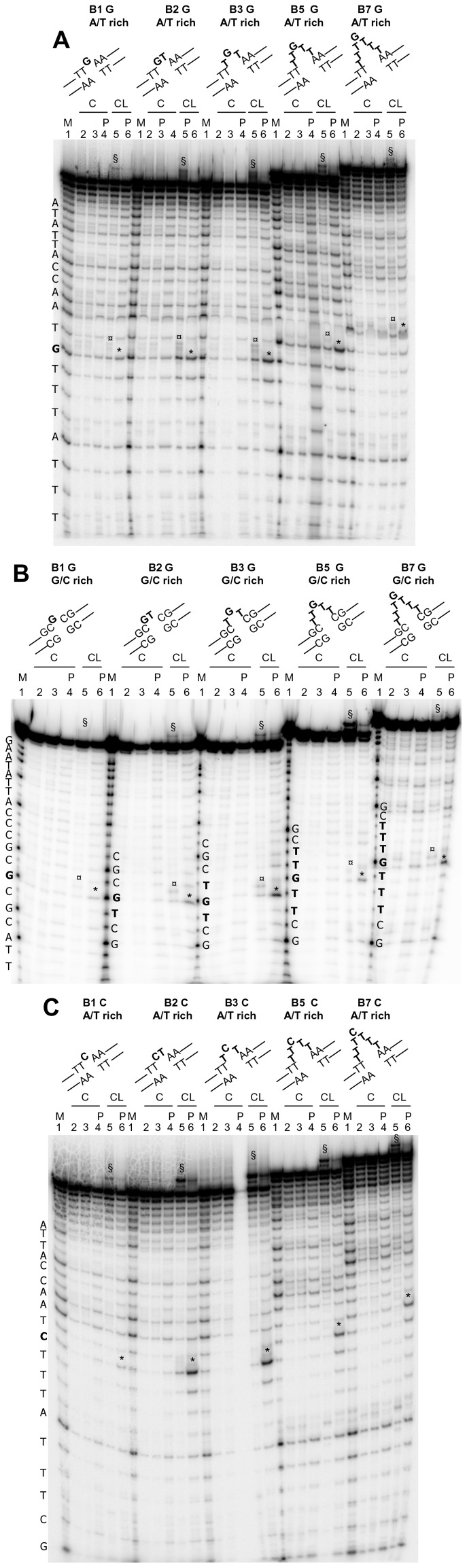
CL footprinting of bulged oligonucleotides. A) Oligonucleotides 1, 6 and 7 were heat denaturated and folded in the presence of the appropriate complementary sequences (1b rev, 1c rev, 1d rev, Table 1) to obtain the bulged G A/T rich oligonucleotides shown above the gel. B) Oligonucleotides 8, 9, 10, 11 and 12 were heat denaturated and folded in the presence of the appropriate complementary sequences (8 rev, 9 rev, 10 rev, Table 1) to the bulged G G/C rich oligonucleotides shown above the gel. C) Oligonucleotides 5, 1, 13, and 14, were heat denaturated and folded in the presence of the appropriate complementary sequences (1b rev, 1c rev, 1d rev, Table 1) to obtain the bulged C A/T rich oligonucleotides shown above the gel. The folded oligonucleotides were incubated with increasing concentrations (50–100 µM) of CL for 24 h at 37°C. After reaction, samples were precipitated and either kept on ice or treated with hot piperidine and lyophilized (samples indicated by the symbol P) and loaded on a 20% denaturing polyacrylamide gel. The symbol § indicates CL/full-length DNA adducts which migrate slower than the full-length DNA. The symbol ¤ indicates bands that correspond to the oligonucleotide alkylated and cleaved by CL. CL is still bound to the cleaved oligonucleotide, thus the cleavage band runs slower than the corresponding band in the Maxam and Gilbert marker lane (M lanes). The symbol * indicates bands that correspond to the oligonucleotide alkylated and cleaved by CL, with loss of CL. Position of alkylation is evinced by comparison of cleavage bands after piperidine treatment and the Maxam and Gilbert marker lane. Oligonucleotide sequences are indicated on the left of the corresponding marker lane (M lanes). Base numbering has been assigned in the 5 prime→3 prime direction.

After reaction with CL, alkylation could be observed before piperidine (slower migrating bands compared to the full-length oligonucleotides and ss G or C marker) and after hot alkali (cleavage bands corresponding to ss G or C) (asterisks, Fig. 4). In the case of bulged Gs flanked by A/T rich regions (Fig. 4A), the amount of cleaved ss G was very poor with 1- and 7-base bulges, while was 3-fold higher with 2-, 3-, 5-base bulges. With bulged Gs flanked by G/C rich ds segments (Fig. 4B), again reaction was extremely poor at 1- and 7-base bulges, incremented by 2-folds with 2- and 5-base bulges, and was maximum with 3-base bulges. With bulged Cs flanked by A/T or G/C rich regions (Fig. 4C and data not shown), the higher cleavage was observed with 3-base bulges, followed by 2-, 5-base bulges; reaction at 7-base bulge was very modest, while no reaction was observed at 1-base bulge (Fig. 1B for summary).

### Hairpins

**Figure 5:**
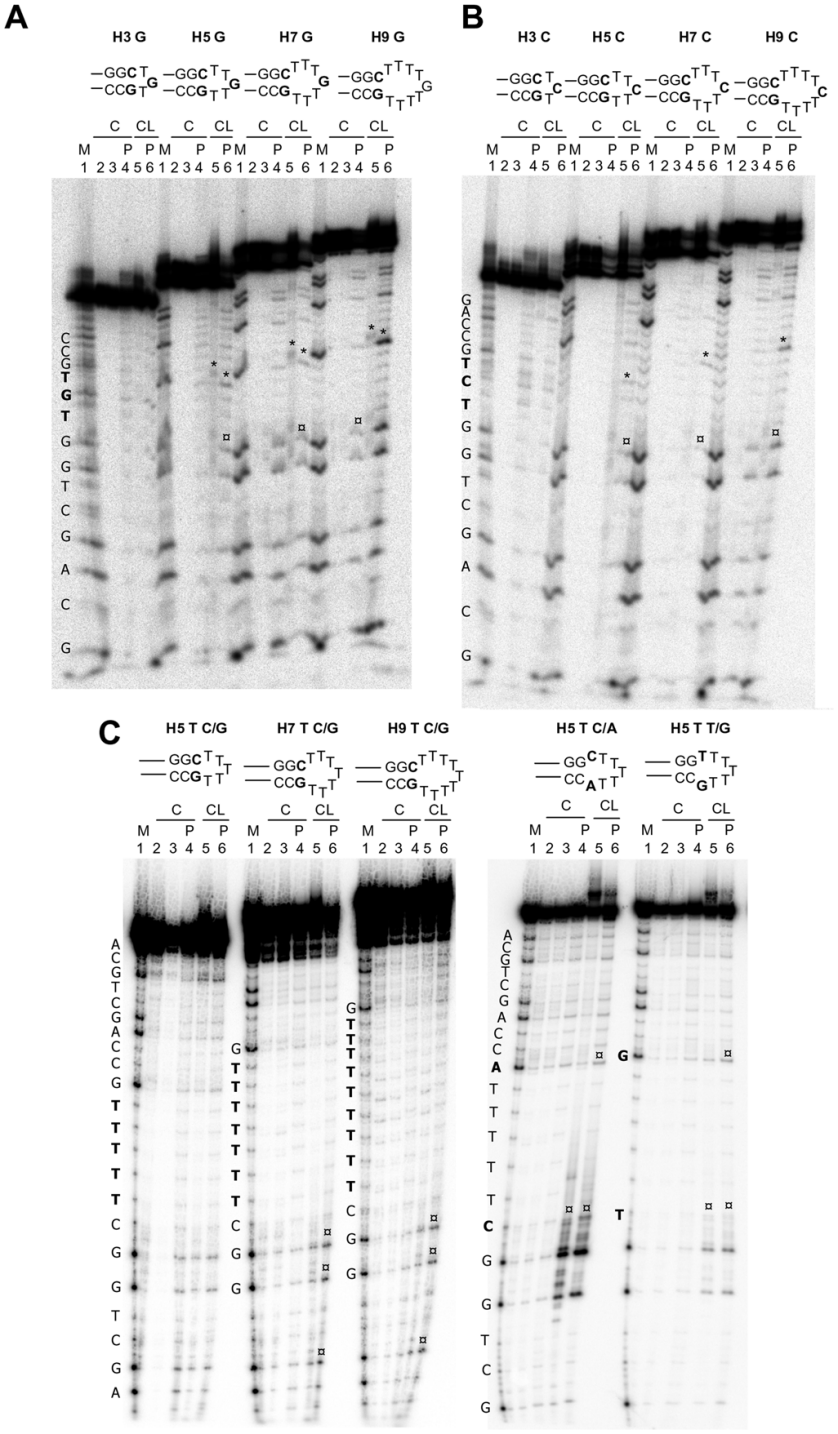
CL footprinting of hairpin oligonucleotides. A) Oligonucleotides 37, 38, 39 and 40, B) oligonucleotides 41, 42, 43 and 44 and C) oligonucleotides 27, 30, 31, 29 and 28 (Table 1) were heat denaturated and folded to obtain the hairpin G, C or T oligonucleotides shown above the gels. The folded oligonucleotides were incubated with CL (100 µM) for 24 h at 37°C. After reaction, samples were precipitated and either kept on ice or treated with hot piperidine and lyophilized (samples indicated by the symbol P) and loaded on a 20% denaturing polyacrylamide gel. The symbol * indicates bands that correspond to the oligonucleotide alkylated and cleaved by CL, without or with loss of CL, at the G or C base exposed in the hairpin region. The symbol ¤ indicates bands that correspond to the oligonucleotide alkylated and cleaved by CL, without or with loss of CL, at bases in the ds stem region of the oligonucleotide. Position of alkylation is evinced by comparison of cleavage bands after piperidine treatment and the Maxam and Gilbert marker lane. Oligonucleotide sequences are indicated on the left of the corresponding marker lane (M lanes). Base numbering has been assigned in the 5 prime→3 prime direction.

Hairpins occur when two regions of the same strand, usually complementary in nucleotide sequence when read in opposite directions, base-pair to form a double helix that ends in an unpaired loop. Hairpins were designed with 3, 5, 7, 9 ss bases. Each loop contained either one G or C flanked by ss T bases, adjacent to G/C rich complementary strands (Table 1 and Fig. 1B). Alkylation at the exposed G or C base was observed in both cases, both prior to and after treatment with hot piperidine, only in loops larger than 3 bases, i.e. with 5, 7 and 9 bases, and CL effects were more evident in the 9 base-loop (asterisks, Fig. 5A and B). Interestingly, however, two adjacent Gs in the ds region were moderately cleaved in the 5-, 7- and 9-base hairpins (¤ symbols, lanes 6, Fig. 5A and B), while their supposedly complementing C bases were not affected by CL alkylation.

Oligonucleotides with loops formed by all Ts were next assayed (Fig. 5C). As expected, no cleavage in the T segment was observed. However, cleavage at the two adjacent Gs in the supposedly ds region was still observed in the 7- and 9-base hairpins (asterisks, lanes 5 and 6, Fig. 5C). The effect of the type of ss bases close to the ds region was thus analysed by assaying oligos with 7-base hairpins formed by CTTTTTA or TTTTTTG ss bases (Fig. 5C). We observed a very strong cleavage at the two adjacent ds Gs in the case of the CTTTTTA hairpin. A less intense effect was detected with the TTTTTTG segment. In this case, however, cleavage was also obtained at the ss G sequence (¤ symbols, lanes 5 and 6, Fig. 5C).

### Long Bulged Regions are Less Sterically Hindered

**Figure 6:**
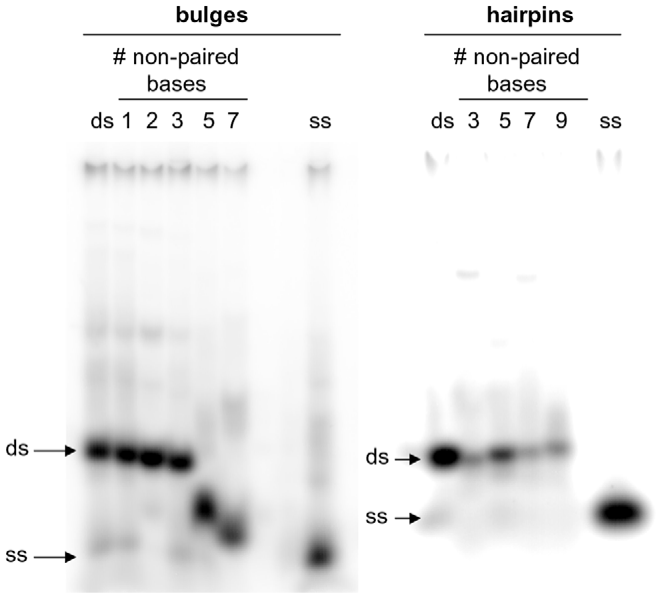
EMSA analysis of bulge and hairpin oligonucleotides. Oligonucleotides 50, 49, 48, 47, 46 and 45 were annealed to the appropriate complementary oligonucleotides (50 rev, 49 rev, 48 rev, 47 rev, 46 rev and 45 rev, Table 1) to form ds, 1-, 2-, 3-, 5-, and 7-base bulged sequences, respectively. Oligonucleotides 54, 53, 52 and 51 were folded to form 3-, 5-, 7-, 9-base hairpin sequences. Ds and ss oligonucleotides with the same length as bulge or hairpin sequences were employed as controls of migration and they are indicated by the arrows aside the gel images.

By testing secondary structures with ss segments of different length we were able to evince that CL reacts at ss region longer than 1 base and in general its reactivity increments with the length of the ss portion. However, one main exception occurred with bulged structures, where the optimal ss length for CL reactivity was 3-bp, whereas lower reactivity was observed either decrementing (1 or 2 bp) or incrementing (5 or 7 bp) the bulge length. To understand why oligonucleotides with more than 3 bulged bases were less susceptible to CL cleavage, bulged sequences of the same overall length but with a different number of bulged bases (Table 1) were tested by electrophoretic mobility shift assay (EMSA). As shown in Fig. 6, electrophoretic mobility of 1-, 2-, 3-bulged oligonucleotides was similar to that of the control ds oligo (compare lanes 1–3 with ds, left side), slightly increasing with the bulge length. Strikingly, the mobility of the 5- and 7-bulged oligonucleotides dramatically incremented and it remained only mildly lower than that of the control ss oligo (compare lanes 5 and 7 with ss, left side). In contrast, the number of non-paired bases in hairpin oligonucleotides only slightly influenced the oligonucleotide electrophoretic mobility, which barely decreased with increasing hairpin length (Fig. 6, right side).

## Discussion and Conclusions

Non-canonical nucleic acid structures have been postulated to mediate protein-nucleic acid interactions and frameshift mutations, some of which may result in a variety of diseases and cancers. Therefore, both recognition and elucidation of the conformation of unusual secondary structures would be of the utmost importance to predict unexpected biological effects generating at the genomic level.

The natural compound CL had been previously shown to be able to detect and induce cleavage in partially ss regions within supercoiled plasmids, whereas it resulted completely inert versus ds nucleic acids [17], [18]. Here we showed that CL could detect the presence of non-paired sequences, when at least one ss non-T base was available, in a number of non-canonical DNA conformations. In addition, the degree of CL reactivity towards DNA bases indicated the accessibility of ss-sites, therefore providing a practical and simple tool to dissect unconventional DNA structures.

In the case of bulges, site accessibility of ss nucleotides varied depending on the length of the bulge itself. By using CL, we were able to demonstrate that the highest base accessibility was obtained when 3-bulged bases were present, indicating that TGT or TCT bulges, with both A/T- or G/C-rich flanking ds sequences, protruded from the double-helix. Accessibility was reduced both in shorter (2 and 1 bases) and, unexpectedly, in longer (5 and 7 bases) bulges. Longer bulges were shown to be less sterically hindered by EMSA; therefore they likely fold back on themselves or stack onto the double-helix, hindering access to reactive groups. These data complement previous analysis on bulge conformations. As a general rule, it was reported that in the case of one-base-bulges, the bulged purines stacked into the duplex [23], [24], [25], [26], whereas bulged pyrimidines were either stacked in or looped out into solution, depending upon the temperature and the flanking sequence [27], [28], [29], [30]. In the case of three- and five-nucleotide DNA bulges, three and five unpaired A bases were found to be mostly stacked into the helix continuously with the flanking DNA and to induce a local kink in the DNA molecule [31], [32]. Unfortunately, only data on bulged A bases were reported for three- and five-base bulges and no detailed structural information is available for different sequences or longer DNA bulges. The data presented here add information on this subject, showing that local folding in the considered sequences occurs only starting from 5-base bulges.

In the case of mismatches, the absence of reactivity towards CL demonstrated that one mismatched base was mostly buried within the double-helix; on the opposite, two mismatched bases was the minimal necessary condition to allow for extra-helical positioning of the non-paired nucleotides. In contrast, when DNA strands were interrupted (nicks), even one non-paired base on the intact strand was effectively exposed and thus available to react with CL. The degree of CL reactivity towards 1 to 3 non-paired nucleosides in nicks did not change, indicating similar exposure of the ss bases. Interestingly, however, some complemented bases close to site of DNA interruption became available for reaction probably due to breathing of the end region of the double-helix.

Opposite to bulges, reactive bases in the loop region of hairpins became more accessible when increasing the length of the loops itself. However, reaction with CL proved that ds bases adjacent to the looped regions did not perfectly pair, thus being accessible for reaction. Availability depended on the nature of the bases within the loop; therefore it is conceivable that non-Watson&Crick base pairing takes place within loop bases. In addition, we demonstrated that hairpins as long as 9 bases did not decrease reactivity towards CL, in contrast to what observed with bulges. Likely, the different relative position of the ss moiety on the ds segment facilitates stacking in the bulges, while hampers folding in the hairpins.

Importantly, in our case we did not find remarkable differences in the reactivity towards ss nucleotides when flanking sequences were either A/T- or G/C-rich, indicating that possible interaction of the extruded ss bases with the adjacent double-helix does not depend on the nature of the bases. Finally, by including either G or C in the ss regions we confirmed the possibility of CL to discriminate between bases, when reactions are visualized both before and after hot piperidine treatment. Differential reactivity towards A can also be achieved, as previously demonstrated [20].

Few high-resolution data are available for non-canonical DNA structures and, in general, data collected so far demonstrated that each sequence determines its own peculiar secondary structure. For this reason is has been difficult to develop compounds that broadly target unusual DNA structures without affecting ds regions. Nakatani’s and Teulade-Fichou’s groups have recently reported compounds able to recognize sequence-specific mismatched DNA and hairpins [13], [33], [34], [35], [36], [37]. No structure-activity relationship can be drawn from these very diverse chemicals, which do not share structural similarities to CL. However, the activities of these compounds and CL are also divergent: the formers target sequence-specific DNA conformations, the latter recognizes all DNA conformations that allow for the presence of single-stranded regions. While the sequence-specific compounds might be useful to treat genetic defects caused by a specific non-canonical DNA conformation, CL can help avoiding the use expensive and cumbersome molecular techniques to detect unusual DNA conformations, which are not readily predictable from sequence data.

CL is a small natural molecule that combines electrophilicity and bulkiness. This modulates the extent of alkylation (and cleavage) of non-canonical DNA conformations. In fact, it allows i) detecting ss regions in a double stranded environment, ii) discriminating between DNA bases within a ss region, iii) reacting to different extents with a given base (except T) as a function of accessibility of the target unpaired nucleotides, iv) easy localization of the target site by sequencing gels.

Since CL is a natural product isolated from a fungus, availability could be a problem. However, total synthesis of CL has been reported [38] and a structural analogue, in which the diterpenoid moiety is replaced by a naphthalene ring while preserving base selectivity and reactivity, can be easily synthesized [18], [19], [20].

Therefore, CL (along with its naphthalene derivative) represents a new valuable tool to localize and monitor unpaired structures in a DNA double helix context.
